# The relations between self-reported perceptions of learning environment, observational learning strategies, and academic outcome

**DOI:** 10.1007/s12528-022-09333-2

**Published:** 2022-08-11

**Authors:** Feifei Han, Robert A. Ellis

**Affiliations:** 1 Institute for Learning Sciences and Teacher Education, Australian Catholic University, Level 4, 229 Elizabeth Street, Brisbane CBD, QLD, 4000, 4000 Brisbane, QLD Australia; 2 Griffith University, 4111 Brisbane, QLD Australia

**Keywords:** Student approaches to learning, Learning analytics, Self-reported perceptions, Observed strategies, Computer science students

## Abstract

This study investigated the relations between students’ self-reported perceptions of the blended learning environment, their observed online learning strategies, and their academic learning outcomes. The participants were 310 undergraduates enrolled in an introductory course on computer systems in an Australian metropolitan university. A Likert-scale questionnaire was used to examine students’ perceptions. The digital traces recorded in a bespoke learning management system were used to detect students’ observed online learning strategies. Using the data mining algorithms, including the Hidden Markov Model and an agglomerative hierarchical sequence clustering, four types of online learning strategies were found. The four strategies not only differed in the number of online learning sessions but also showed differences in the proportional distribution with regard to different online learning behaviors. A one-way ANOVA revealed that students adopting different online learning strategies differed significantly on their final course marks. Students who employed intensive theory application strategy achieved the highest whereas those used weak reading and weak theory application scored the lowest. The results of a cross-tabulation showed that the four types of observed online learning strategies were significantly associated with the better and poorer perceptions of the blended learning environment. Specially, amongst students who adopted the intensive theory application strategy, the proportion of students who self-reported better perceptions was significantly higher than those reporting poorer perceptions. In contrast, amongst students using the weak reading and weak theory application strategy, the proportion of students having poorer perceptions was significantly higher than those holding better perceptions.

## Introduction

The coronavirus pandemic (COVID-19) emergency has required higher education learning and teaching around the world to rapidly respond, in particular, redeploying more learning and teaching activities to virtual learning spaces to promote physical distancing. As a result, the face-to-face courses have been delivered either as blended courses or as purely online courses (Tang, Chen, Law, Wu, & Lau, 2021). In such urgent transformation, it is important to examine the relations of how learners perceive their learning environment (their perceptions) and how they approach the learning (their learning strategies). While past research has indicated the importance of students’ perceptions of the learning environment and has examined the relations between the perceptions, learning strategies, as well as academic learning outcomes, much of the research is based on a single source of evidence, typically self-reports (Guo, 2018; Guo et al., 2017; Lizzio et al., 2002; Wilson & Fowler, 2005). To improve the robustness of the findings of such research, it is valuable to triangulate it with the observational measures of learning strategies, such as detailed digital traces recorded in the learning management system (LMS). The combined sources of the evidence will provide a more holistic understanding of what students actually do (reflected by the observational measures) and why they do it (reflected by the self-reported measures) (Ellis et al., 2016; Han & Ellis, 2020a). The current study aims to address this purpose by investigating students’ self-reported perceptions of the blended learning environment and their observed online learning strategies by drawing on Student Approaches to Learning (SAL) research and Learning Analytics research. The following section will review the relevant literature from the two areas.

## Literature review

### Relevant student approaches to learning research

**Fig. 1:**
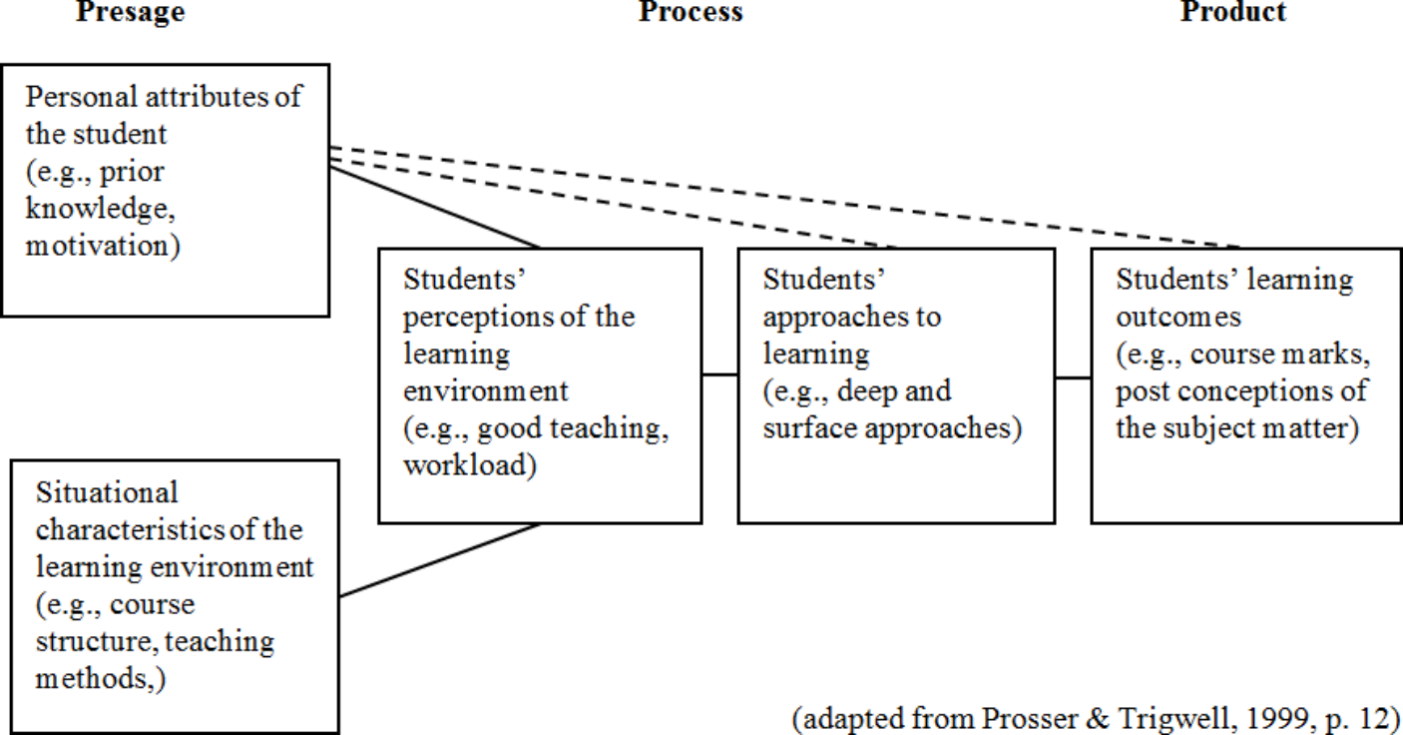
shows that students’ prior experiences of learning and the situational context are part of the Presage stage (Prosser & Trigwell, 1999). In the Process stage, students’ approaches to learning and their perceptions of the learning context and environment (e.g., perceptions of the quality of teaching practice, assessment methods, and study workload) are two important factors (Lizzio et al., 2002). Students’ learning outcomes is located in the Product stage, and may include assessment marks and/or students’ understanding of the key concepts in the subject matter

Student Approaches to Learning (SAL) research is a recognised guiding framework for the enhancement and assessment of the quality of learning in higher education (Biggs & Tang, 2011; Trigwell & Prosser, 2020). This area of research has shown that students’ prior experiences of learning, the departmental context, and students’ perceptions of the current learning contexts, are all closely related to their learning processes and the quality of learning outcomes (Biggs & Tang, 2011; Trigwell & Prosser, 2020). Research in this area mostly uses self-reported measures, such as surveys and interviews, to examine the key aspects in the students’ experiences of learning (Ramsden, 2003). To describe the relations of these key aspects, Biggs (1989) proposed a Presage-Process-Product model (known as 3P model), which has later been refined by Prosser & Trigwell (1999) and is visually represented in Fig. 1.

The elements included in the 3P model represent a relational system, which denotes that these elements are not linear nor are bound by chains of causality, but rather they coexist simultaneously. The Presage factors can be closely related to the learning outcomes in some contexts and they can also have indirect relations with the learning outcomes via the elements in the Process, which serve as mediators (Prosser & Trigwell, 2017; Trigwell & Prosser, 2020).

Previous SAL research on students’ perceptions has demonstrated that when students perceive the teaching of a high quality and being well organised, and the assessment tasks fit the learning objectives, they are more likely to use deep approaches and strategies in learning. In contrast, when students see teaching goals being unclear and unfocused, workload being too heavy, assessment tasks inappropriate, and a lack of teacher-student interactions, they tend to adopt surface approaches and strategies (Crawford et al., 1998; Lizzio et al., 2002; Wilson & Fowler, 2005).

In blended course designs, which require students to shift back and forth between face-to-face and online modes, research has reported that students who perceive that face-to-face and online components of learning and teaching are well integrated, adopt more deep approaches to learning but less surface approaches. In contrast, those who see that the face-to-face and online learning are fragmented and unaligned, are more likely to approach learning at a surface level (Ellis & Bliuc, 2019; Han & Ellis, 2019). Furthermore, logical associations between perceptions, learning approaches and strategies, and the learning outcomes, have also been found, across a number of academic disciplines, such as business (Han & Ellis, 2019), sciences (Ellis & Bliuc, 2019), social sciences (Ellis et al., 2020), and engineering (Ellis et al., 2016). Students having relatively better perceptions and adopting deep approaches are often found to have higher academic achievement than their peers holding poorer perceptions and using surface approaches.

These previous SAL investigations have predominantly employed self-reported instruments and data to examine these relations (Ellis & Bliuc, 2019; Ellis et al., 2017; Han & Ellis, 2019). While the self-reporting has the merit to capture students’ perceptions and intentions, and help explicate the reasons behind their decisions on their learning actions, behaviors, and strategies (Zhou & Winnie, 2012); the self-reported evidence in objectively representing what and how students learn in reality have been questioned (Hadwin et al., 2007). In addition, compared with the observational measures of students’ online learning strategies, it is relatively more difficult for self-reported data to capture the complex and dynamic natures of students’ online learning behaviors. In the current study, observational measures will be used to represent students’ online learning strategies.

### Relevant Learning Analytics Research

In the past decade, the development of educational technology has produced prolific learning analytic studies, which emphasize on the capacity to collect detailed digital traces of students’ interactions with a variety of online learning resources and activities. The digital trace type of data, also known as the observational data have the advantage of offering descriptions of students’ learning behaviors and strategies relatively more objectively and in a more granular details than using self-reported methods (Siemens, 2013). The observational analytic data when combined with students’ demographic information have been increasingly used in various domains in higher education sector, such as advising students’ career choice (Bettinger & Baker, 2014); detecting at risk students to improve retention (Krumm et al., 2014); providing personalised feedback (Gibson, Aitken, Sándor, Buckingham Shum, Tsingos-Lucas et al., 2017); identifying patterns of learning tactics and strategies (Chen et al., 2017); facilitating collaborative learning (Kaendler et al., 2015); monitoring students’ affect in learning (Ocumpaugh et al., 2014); and predicting their academic learning outcomes (Romero et al., 2013).

However, being solely dependent on the observational digital traces and overly relying on sets of quantitative numbers has the danger of producing meaningless results and may result in reduced insights in interpretation due to a lack of proper guidance from theories, hence limiting the usefulness of the analytic data to locate barriers of learning, to offer ideas for pedagogical reforms, and to provide guidance for learning designs (Buckingham Shum & Crick, 2012).

To address the drawbacks of an overly empirical approach to learning analytics research, proposals have been put forward to use a more holistic approach to designing research and to guiding data analysis and modelling in order to improve the interpretability of the quantitative results derived from the observational digital traces (Gašević et al., 2015; Toetenel & Rienties, 2016). As a result, an increasing number of studies have combined observational and self-reported measures to examine students’ learning (Lockyer, Heathcote, & Dawson, 2013). This combined approach allows students’ learning behaviors and strategies to be interpreted through a more holistic assessment (Reimann et al., 2014).

In adopting a combined approach comprising self-reported and observational measures and data, research has been conducted to achieve two main purposes. The first purpose is to increase the explanatory power of the prediction of the learning outcomes by using different types of data. The majority of the existing research for this purpose has demonstrated that an inclusion of the observational measures of students’ learning behaviors have significantly improved the prediction of the learning outcomes than by using self-reporting alone Han & Ellis 2020a; Rodríguez-Triana et al., 2015; Tempelaar, Rienties & Giesbers, 2018). For instance, Pardo et al., (2017) reported that adding the frequency of students’ interactions with the online learning activities explained an extra 25.00% of variance in students’ course marks than using their reported use of self-regulated learning strategies alone. Similarly, Ellis et al., (2017) also found that by adding the quantity of students’ online participation in the regression model has significantly increased the variance explained in students’ academic performance than merely using students’ reported learning approaches.

Another aim of the studies adopting a combined approach is to investigate the extent to which the self-reported and observational measures of students’ learning are consistent and aligned with each other (Rodríguez-Triana et al., 2015). Research in this category has examined the relations between the observed online learning behaviors and various self-reported measures involved in students’ learning processes, such as self-efficacy and anxiety (Pardo et al., 2017); learning orientations (Han & Ellis, 2021; Han et al., 2020); learning motives (Gašević et al., 2017); learning engagement (Ober, Hong, Rebouças-Ju, Carter, Liu et al., 2021); achievement goal orientations (Sun & Xie, 2020); and effort (Li et al., 2020). However, the research evidence between the self-reported and observational measures has not always been coherent.

For instance, drawing a self-regulated learning perspective, Pardo et al., (2017) found that Australian university students who self-reported having higher intrinsic motivation were also found to view the video course contents more frequently that their peers who reported a lower level of intrinsic motivation. In another study with 320 American high school students, however, Ober et al., (2021) found that students’ online learning behaviors measured by a number of indicators, including frequencies of their assignment completion and results checking, and the average duration of the computer sessions students produced, were largely uncorrelated with their responses to a learning engagement questionnaire. Clearly further research is required to investigate the extent of consistency between the self-reported and observational measures of students’ learning.

### The current study and research questions

The current study will investigate the relation between students’ self-reported perceptions of the blended learning environment and their academic learning outcomes on one hand; the relation between students’ observed online learning strategies and their academic learning outcomes on the other hand. It will then examine the relation between students’ self-reported perceptions of the blended learning environment and their observed online learning strategies. Specifically, the study addressed three research questions:

1. What is the relation between students’ self-reported perceptions of the blended learning environment and their academic learning outcomes?
2. What is the relation between students’ observed online learning strategies and their academic learning outcomes?
3. What is the relation between students’ self-reported perceptions of the blended learning environment and their observed online learning strategies?

## Method

### Participants and the research context

The participants of the study were 310 undergraduates (aged between 17 and 31, *M* = 19.67, *SD* = 2.05). They were all enrolled in a first-year introductory course on computer systems, which was a blended course required students to attend face-to-face lectures and tutorials and to interact online. The online learning, which was held in a bespoke LMS, consisted of five major online learning resources: printed course contents, video course contents, problem-solving sequences, multiple-choice questions of testing the key concepts, and a dashboard for feedback and online learning progression. The bespoke LMS was designed by the course coordinator and had been adopted in this course for many years. The reason for using a bespoke LMS rather than a commercial LMS was it had more advanced learning analytic functions, such as recording the exact time students’ logon and logoff time and the timestamps of sequences of students’ online learning bebaviors.

### Data and Instruments

**Self-reported perceptions collected by a questionnaire.** Students’ self-reported perceptions of the blended learning environment were collected using a 5-point Likert-scale questionnaire, which consisted of two scales: (1) perceptions of the integration between face-to-face and online learning, which assessed students’ perceived level of how face-to-face and online learning in the course are integrated (7 items, α = 0.86); (2) perceptions of online contributions, which examined students’ perceptions of how they valued online learning (6 items, α = 0.87). The questionnaire was used in previous SAL research (Ellis & Bliuc, 2019; Han et al., 2010) and the validity and reliability have been reported in (Han & Ellis, 2020b).

**Observed online learning behaviors recorded by the bespoke LMS.** The observed online learning behaviors were extracted from the LMS using the analytic functions. The LMS recorded students’ identifiers (represented by unique identification numbers to anonymize the names of the students), the type of online learning behaviors, and the timestamps of sequences of online learning behaviors. An online learning behavior was defined as a click on a type of online learning resource. Hence, students’ clicks on the five different types of online learning resources represented five different types of online learning behaviors, namely: reading behaviors (reading the printed course contents); watching behaviors (watching the video course contents), theoretical testing behaviors (doing multiple-choice questions of testing the key concepts), theory application behaviors (applying theories in problem-solving sequences),and study monitoring behaviors (viewing the dashboard for feedback and online learning progression).

**The academic learning outcome.** The academic learning outcome was students’ course mark consisted of students’ lecture and tutorial attendance and a close-book examination in the multiple-choice questions format. The examination assessed students’ understanding of key theoretical points and their abilities to ultilise theories to solve practical problems.

### Ethics Considerations of the Data Collection

The ethics guidelines were strictly followed to recruit the participants and to collect the data. Before the study, all the potential participants were informed about the purposes of the study. In the Participant Information Statement, it was clearly explained to students that their participation was entirely voluntary and their decisions on participation or not would by no means impact on their course marks as the teaching staff in the course had no access to the data. They were also ensured that their identification would be anonymized, and all the information collected would be used solely for the research purposes. Students were required to sign a written consent form should they wish to participate.

### Data Analysis

To answer the first research question – the relation between students’ self-reported perceptions of the blended learning environment and the academic learning outcome, the *Mean* scores of the two perceptions scales were used to divide students into two groups of having better or poorer perceptions. A one-way ANOVA on students’ course marks between those having better and poorer perceptions was performed.

To provide an answer to the second research question – the relation between students’ observed online learning strategies and their academic learning outcome, the algorithm of the Hidden Markov Model (HMM) was applied on the sequences of students’ online learning sessions. One online learning session was defined as continuous online learning behaviors with less than 30-minute breaks. One online learning sessions may consist of varying number of the timestamped online learning behaviors. The HMMs transformed each online learning session into an online learning state, which was represented by a predominant online learning behavior (but might comprise more than online learning behaviors). After the procedure of HMM transformations, the chains of the transformed online learning states were subjected to an agglomerative hierarchical sequence clustering analysis to derive distinct patterns of students’ online learning strategies. Using the online learning strategies as a between-subjects variable, a one-way ANOVA on students’ course marks was conducted.

For the last research question – the relation between students’ self-reported perceptions of the blended learning environment and their observed online learning strategies, a cross-tabulation was conducted between groups by perceptions and by the online learning strategies.

## Results

### The relations between self-reported perceptions of the blended learning environment and the academic learning outcome

The result of the one-way ANOVA shows that on students’ reporting better and poorer perceptions differed significantly on their academic performance in the course: *F* (1, 308) = 8.33, *p* < .01, η^2^ = 0.02. Students who had higher ratings of the blended learning environment (*M* = 88.58, *SD* = 17.24) obtained significantly higher course marks than those with lower ratings of the blended learning environment (*M* = 83.11, *SD* = 16.14).

### The relations between the observed online learning strategies and the academic learning outcome

Using the Akaike Information Criterion (AIC) and the Bayesian Information Criterion (BIC), the HMMs identified three states of the online learning sessions, which were described below:

- reading states predominantly consisted of reading, and few study monitoring;
- theory application states predominantly consisted of applying theories to solve practical problems, few reading and watching;
- theoretical testing states predominantly consisted of theoretical testing, few reading and study monitoring.

**Fig. 2:**
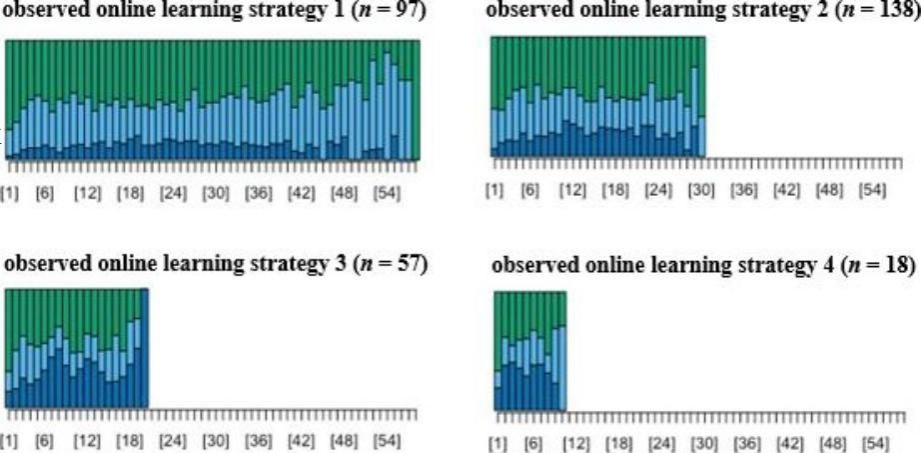
The four observed online learning strategies. Notes: green = reading states, light blue = theory application states, dark blue = theoretical testing states

Using the above three states, an agglomerative hierarchical sequence clustering analysis was performed. To select the optimal number of clusters, dendrograms were used to identify the most plausible segmentations.

of the tree structure (Kassambara, 2017). Four clusters were retained, with each cluster representing a distinct observed online learning strategy. The four online learning strategies are visually presented in Fig. 2.

In Fig. 2, each point in the X axis is a transformed HMM state of the corresponding online learning session. The Y axis shows the proportional distribution of the HMM states. As shown in Fig. 2, the students in the four clusters not only differed by the number of the learning states, but also differed on the proportional distribution of the types of the states. In general, the proportions of the reading states were similar amongst the first three types of the observed online learning strategies, which were all higher than that in the observed online learning strategy 4. The differences were mainly in the proportional distribution of the theory application states and theoretical testing states.

- observed online learning strategy 1 (n = 97) – intensive theory application: Students adopting strategy 1 had high percentage of theory application states but low percentage of theoretical testing states. These students also had the most online learning sessions.
- observed online learning strategy 2 (n = 138) – moderate theory application: Students adopting strategy 2 had moderate percentage of theory application states but low percentage of theoretical testing states. These students had the second most online learning sessions.
- observed online learning strategy 3 (n = 57) – weak theory application and moderate theoretical testing: Students adopting strategy 3 showed features of having low percentage of theory application states but moderate theoretical learning. The online learning sessions of this group of students ranked the third.
- observed online learning strategy 4 (n = 18) – weak reading and weak theory application: Students adopting strategy 4 had low percentages of both the reading states and the theory application states. The students in this cluster had the least online learning sessions.

The results of the one-way ANOVA by students’ observed online learning strategies showed that their learning outcomes significantly differed: *F* (3, 306) = 36.75, *p* < .01, η^2^ = 0.27. The post-hoc tests for pair-wise comparison were conducted. Due to the unequal sample size between groups, Gabriel’s post-hoc test was selected and the results of the pairwise comparison are displayed in Table 1.

Table 1 shows that students who adopted the intensive theory application strategy (strategy 1) (*M* = 94.49, *SD* = 11.56) obtained the highest course marks than the students using the other three types of online learning strategies. Students using the moderate theory application strategy (strategy 2) had the second highest marks (*M* = 87.18, *SD* = 15.50), followed by those with the weak theory application and moderate theoretical testing strategy (strategy 3) (*M* = 75.71, *SD* = 16.83). The students employing the weak reading and weak theory application strategy (strategy 4) obtained the lowest marks (*M* = 62.31, *SD* = 13.70).

**Table 1:** Results of the post-hoc analyses of the academic learning outcome

observed online learning strategies	*M*	*SD*	pairwise
intensive theory application (strategy 1)	94.49	11.56	1 > 2
			1 > 3
			1 > 4
moderate theory application (strategy 2)	87.18	15.50	2 < 1
			2 > 3
			2 > 4
weak theory application and moderate theoretical testing (strategy 3)	75.71	16.83	3 < 1
			3 < 2
			3 > 4
weak reading and weak theory application (strategy 4)	62.31	13.70	4 < 1
			4 < 2
			4 < 3

### The relation between the self-reported perceptions of the blended learning environment and the observed online learning strategies

The results of the 2 (two groups of students having better vs. poorer perceptions) x 4 (four groups of students using four online learning strategies) cross-tabulation was significant χ² (3) = 8.76, *p* < .05, φ = 0.03. The two-proportion *z*-tests displayed in Table 2 show that amongst 97 students who adopted the intensive theory application strategy (strategy 1), the proportion of students self-reporting better perceptions of the blended learning environment (59.80%) was significantly higher than the proportion of those reporting poorer perceptions (40.20%). In contrast, amongst 18 students who used weak the reading and weak theory application (strategy 4), the proportion of students having poorer perceptions of the blended learning environment (72.20%) was significantly higher than the proportion of students holding better perceptions (27.80%).

**Table 2:** Results of the cross-tabulation

intensive online learning strategies	count % within perceptions	poorer perceptions	better perceptions	total
moderate theory application (strategy 1)	count	39_a_	58_b_	97
	% within perceptions	40.20%	59.80%	100.00%
moderate theory application (strategy 2)	count	66_a_	72_a_	138
	% within perceptions	47.80%	52.20%	100.00%
weak theory application and moderate theoretical testing (strategy 3)	count	33_a_	24_a_	57
	% within perceptions	57.90%	42.10%	100.00%
weak reading and weak theory application (strategy 4)	count	13_a_	5_b_	18
	% within perceptions	72.20%	27.80%	100.00%
total	count	151	159	310
	% within perceptions	48.70%	51.30%	100.00%

*Notes*: Different subscript letters denote the categories of the observed online learning strategies whose column proportions differed significantly from each other at the 0.05 level

## Discussion

This study examined the relations between students’ self-reported perceptions of the blended learning environment, their observed online learning strategies, and their academic learning outcome. Similar to the previous research findings (Guo, 2018; Guo et al., 2017; Ellis & Bliuc, 2019; Han & Ellis, 2020a), our study also found that students who had better perceptions of the learning environment (in this context, those who perceived that the online learning part was well blended with the face-to-face part in the course, and appraised the online contributions) tended to achieve better academic performance in the course.

Different from the methods used in the previous studies, which used self-reports to measure students’ learning strategies and/or approaches (Ellis & Bliuc, 2019; Ellis et al., 2017), we employed the digital traces left in the LMS – a more objective measure to represent students’ online learning strategies. The data mining techniques detected four types of the online learning strategies, which not only differed in terms of the number of the online learning sessions (how much students learned online), but also varied with regard to the proportional distribution of the different online learning behaviors (how they learned online). Similar to the results reported in Han & Ellis (2017), our results also indicated that the more the students participated in the online learning, the higher course marks they obtained. In addition, the results also suggested that the students who interacted more with theory application resource tended to achieve better academic learning outcomes; as students adopting online learning strategy 1 and 2 had significantly higher course marks than those using online learning strategy 3 and 4. One possible interpretation of the results could be that engagement with theory application might represent a deeper level of learning than merely testing theoretical concepts, as solving sequences of problems not only required a thorough understanding of theories, but also the abilities to apply theories in tackling problems and issues in real life. This meant that students might need to draw on relevant theories, apply formula, use mathematical methods, and build models in order to successfully complete the theory application tasks. Such findings seemed to align with previous SAL findings, which consistently reported association between the deep strategies reported by students and better learning outcomes (Trigwell & Prosser, 2020). The observed results from our study added more objective evidence and offered some triangulations for the previous self-reported research evidence.

Our research results also share some similarities with the learning analytics studies on detecting students’ online learning tactics and strategies. These studies reported that students differed in terms of how much they were engaged with different types of the online learning activities; and such differences on approaching a certain type or a combination of certain types of learning activities also tend to relate to their academic performance (Fincham et al., 2018; Jovanović et al., 2017). However, both the current study and the existing studies did not provide a clear answer to the question of whether how much students learned online (e.g., the total number of the online learning sessions), or how they learned online (e.g., the proportional distributions of the different online learning behaviors) or both of the two factors, are related to students’ academic performance. This question needs to be answered by clustering students using the criterion of either the quantity of the online learning or the proportional distributions of different types of the online learning behaviors.

With regard to the relation between the self-reported perceptions of the blended learning environment and the observed online learning strategies, the study found a significant association between the better and poorer perceptions and the patterns of the observed strategies. In particular, of the students using the intensive theory application strategy (also had the highest course marks), a higher proportion of them perceived the blended learning environment more positively; whereas of those employing the weak reading and weak theory application strategy (also had the lowest course marks), a higher proportion of them had more negative perceptions towards the blended learning environment.

These findings seem to be consistent with the studies employing the self-reported methods to examine the relations between the perceptions of learning environment and the learning strategies/approaches (Ellis & Bliuc, 2019; Guo, 2018; Han & Ellis, 2020a). The results of our study not only confirm and triangulate previous self-reported findings, the digital trace measures also offer much more detailed descriptions about the online learning behaviours than what can captured by using questionnaires. Notwithstanding such merit, cautions still need to be taken when comparing the results from the self-reporting methods and the observational methods, as the learning strategies measured by self-reports often include the information about students’ motives and intents of adopting certain types of strategies (why question). Therefore, the online learning strategies measured by observation can only be used to approximate the strategies and approaches measured by self-reports.

## Limitations and future research direction

A number of limitations of the study need to be pointed out in order to inform future research. First, as mentioned, the clustering of students’ observed online learning strategies did not distinguish clearly between the number of the online learning sessions and the proportional distribution of the online learning states. Future research should purposely address some of the unanswered questions brought up by this limitation, such as whether the number of the online learning sessions, or the proportional distribution of the online learning activities, are related to students’ perceptions of the learning environment and their academic performance. Second, the research was conducted with students only from one academic discipline – computer science. To examine if there are disciplinary variations of the results, the similar research design with students from other academic disciplines should be conducted in the future. Furthermore, the self-reported data only measured students’ perceptions of the blended learning environment. The SAL research has indicated that students’ personal attributes, their prior knowledge, and their motivation in the learning context, are all related to their perceptions, strategies, and academic learning outcomes (Trigwell et al., 2013). New studies which address these issues in this area will help push the field onwards.
